# Inhibition of the polyol pathway by *Ducrosia anethifolia* extract: plausible implications for diabetic retinopathy treatment

**DOI:** 10.3389/fphar.2024.1513967

**Published:** 2024-12-23

**Authors:** Saheem Ahmad, Mohammad Faizan Ali Ahmad, Saif Khan, Sultan Alouffi, Mahvish Khan, Mohd Wajid Ali Khan, Irfan Ahmad Ansari

**Affiliations:** ^1^ Department of Medical Laboratory Sciences, College of Applied Medical Sciences, University of Hail, Hail, Saudi Arabia; ^2^ Department of Biosciences, Integral University, Lucknow, India; ^3^ Department of Basic Dental and Medical Sciences, College of Dentistry, University of Hail, Hail, Saudi Arabia; ^4^ Department of Biology, College of Science, University of Hail, Hail, Saudi Arabia; ^5^ Department of Chemistry, College of Science, University of Hail, Hail, Saudi Arabia

**Keywords:** diabetic retinopathy, polyol pathway, advanced glycation end products (AGEs), aldose reductase (AR), sorbitol dehydrogenase (SD)

## Abstract

**Introduction:**

Diabetic retinopathy is a significant microvascular disorder and the leading cause of vision impairment in working-age individuals. Hyperglycemia triggers retinal damage through mechanisms such as the polyol pathway and the accumulation of advanced glycation end products (AGEs). Inhibiting key enzymes in this pathway, aldose reductase (AR) and sorbitol dehydrogenase (SD), alongside preventing AGE formation, may offer therapeutic strategies for diabetic retinopathy and other vascular complications. This study investigates the ability of *Ducrosia anethifolia*, an Arabian plant, to inhibit AR and SD enzymes.

**Methods:**

Methanolic extracts of the plant were tested in enzyme assays and further analyzed using Lineweaver-Burk plots for kinetic insights. Additionally, the effects on AGE production and sorbitol accumulation in red blood cells were evaluated.

**Results:**

The methanolic extract showed strong inhibitory effects on AR (IC50: 69.41 ± 3.59 μg/mL) and SD (IC50: 31.11 ± 5.58 μg/mL), acting through a mixed-inhibition mechanism. It also significantly reduced sorbitol accumulation and AGE formation.

**Discussion:**

These findings suggest that the extract’s inhibition of the polyol pathway enzymes is due to its phytochemical content. Further isolation and identification of these active compounds could provide valuable insights for developing future pharmaceutical treatments for diabetic retinopathy.

## Introduction

Diabetic retinopathy, a significant microvascular disorder, is the primary factor contributing to vision impairment (Parveen et al., 2018). Polyol, protein kinase C, and hexosamine pathways, as well as the accumulation of advanced glycation end-products (AGEs), have been identified as potential mechanisms responsible for retinal microvascular damage caused by hyperglycemia (Simó et al., 2022; Wang and Lo, 2018). In hyperglycemic conditions, where excess glucose accumulates, the polyol pathway becomes increasingly active, converting glucose to sorbitol and fructose. This shift in glucose metabolism contributes to cellular stress and is implicated in the pathogenesis of diabetic complications such as retinopathy (Dănilă et al., 2024). Polyol pathway is a two-step reaction, initially facilitated by aldose reductase (AR) and then by sorbitol dehydrogenase (SD), where glucose is enzymatically broken down to sorbitol and then further transformed to fructose (Jannapureddy et al., 2021). Additionally, a persistent hyperglycaemic milieu leads to increased synthesis of AGEs (Ahmad et al., 2024; Akhter et al., 2024; Nabi et al., 2020). During the formation of AGEs proteins or lipo-proteins and in some cases, DNA reacts with the reducing sugars such as glucose, fructose, glyoxal and methylglyoxal leading to the cascade of reactions leading to AGEs formation and accumulation (Ahmad et al., 2011; Khanam et al., 2024; Ishrat et al., 2024; Khan et al., 2024; Shahab et al., 2017; Jabir et al., 2018). Increased intracellular concentration of sorbitol and fructose has been observed under hyperglycaemic environment (Rungratanawanich et al., 2021). According to Julius et al. (2022), fructose has a high affinity for the amino groups of proteins, leading to the non-enzymatic formation of early glycation products. These products then undergo a series of intricate processes to produce fluorescent molecules known as AGEs.

Vascular problems linked to diabetes have been ascribed to the abnormal accumulation of sorbitol in the cells (Taslimi et al., 2018). Aggregation of sorbitol in the eye lens leads to the development of sugar cataracts (Chang et al., 2019). The rapid generation and accumulation of AGEs in several organs leads to oxidative stress and has detrimental impacts on several cellular processes (Rungratanawanich et al., 2021). Hence, by inhibiting AR and SD enzymes, and preventing the formation of AGEs, it is possible to mitigate the risk of cataract development, diabetic retinopathy, and other microvascular problems associated with diabetes (Homme et al., 2018). Furthermore, the identification and assessment of precise and potent inhibitors of AR and SD enzymes would be valuable in the advancement of efficacious treatments for various diabetic vascular challenges, such as diabetic retinopathy.

Given its historical use as an antidiabetic plant and the presence of several significant bioactive components, *Ducrosia anethifolia* presents a promising option for the treatment of diabetic retinopathy (Shayganfar et al., 2022). The plant *Ducrosia anethifolia* Boiss is a drought-tolerant species that thrives extensively in the desert of Saudi Arabia. This botanical specimen belongs to the Apiaceae family and possesses therapeutic attributes (Akbar and Arezoo, 2017). The aerial portions of *Ducrosia anethifolia* have been traditionally utilised in Asian nations including Afghanistan, Iran, Iraq, Pakistan, and Lebanon for the treatment of headaches, backaches, and colic pain (Mottaghipisheh et al., 2020; Haghi et al., 2004). The recognised effects of the aerial components of *Ducrosia anethifolia* on the central nervous system include its use as an anxiolytic, antidepressant, and treatment for insomnia (Mottaghipisheh et al., 2020). The published research indicates that the crude extract of *Ducrosia anethifolia* is associated with a diverse range of medicinal advantages. The advantages highlighted in the study by Shahabipour et al. (2013) encompassed anti-diabetic, anti-microbial, anti-radical scavenging, anti-inflammatory, anti-cancer, ant locomotor, and anxiolytic capabilities (Shahabipour et al., 2013). Few previous studies have identified several bioactive compounds including flavonoids, coumarins, phenolic acids, and terpenoids, which are commonly recognized for their roles in mitigating oxidative stress and inflammation (Almuhanna, 2024; Sarabandi et al., 2022; Mottaghipisheh et al., 2020; Elsharkawy et al., 2019; Arbabi et al., 2018; Mottaghipisheh et al., 2014). While there have been few prior studies on the antidiabetic properties of *Ducrosia anethifolia*, there is currently no data to date about its impact on enzymes associated with diabetic complications like diabetic retinopathy (Syed et al., 2022; Shalaby et al., 2014). Hence, the aim of this work was to assess the potential of *Ducrosia anethifolia* to regulate enzymes related to diabetic problems.

## Materials and methods

### Chemicals

Sigma-Aldrich provided human recombinant AR, DL-glyceraldehyde, reduced NADPH, quercetin, DMSO, sorbitol, SD, and oxidised NAD^+^. Each of the other chemicals and solvents used were of analytical grade.

### Plant sample collection and preparation of extract

*Ducrosia anethifolia* plants were gathered from the Al-Nafud Al-Kabir desert in Hail, Saudi Arabia. A taxonomist positively recognised the plants and a voucher specimen was subsequently submitted to the Herbarium of the University of Hail. Once dusts were eliminated by rinsing with Millipore water, fresh leaves were gathered and dried naturally. The leaves were pulverised using a mechanical grinder to obtain a fine powder. Trimbles were then made by weighing 30 gm of this powder. The thimble was inserted into a Soxhlet extractor and then 250 mL of 90% methanol was added. The extraction was conducted continuously for 5–6 h at a temperature ranging from 50° to 60°C. The plant extract underwent filtration, vacuum drying, and was then stored in a glass bottle at a temperature of 4°C for future use.

### Assessment of AR inhibition

The AR inhibition assay was conducted following published procedures with minor changes (Kazeem et al., 2020; Fatmawati et al., 2015). The reaction mixture consisted of 0.15 mM NADPH, 10 mM DL-glyceraldehyde, 5 μL of AR at a concentration of 100 μg/mL, and 100 μL of methanolic leaf extract from *Ducrosia anethifolia* (12.5–200 μg/mL in DMSO less than 1%) or DMSO alone. The total volume of the combination was 1.0 mL of 100 mM sodium phosphate buffer at a pH of 6.2. Once the reaction solutions were pre-incubated at 25°C for 5 min, the enzyme was added to initiate the reaction. The change in absorbance was then recorded at 340 nm for 3 min using a BioSpectrometer (Eppendorf, United States). Quercetin served as the positive control. To determine the percentage inhibition of AR, the following equation was used:

$$\%\text{ Inhibition}=\left[\left(\Delta \text{Abs}\;\text{control}-\Delta \text{Abs extract}\right)÷\;\Delta \text{Abs control}\right]\times 100$$

where, ΔAbs_control_ and ΔAbs_extract_ represent change in the absorbance of control (DMSO only) and leaf extract, respectively.

### Analysis of the AR kinetics

The suppression mechanism of AR by methanolic leaf extract was carried out following the previously described method with slight modifications (Kazeem et al., 2020; Dongare et al., 2012). Each set of tubes contained a reaction mixture consisting of 0.15 mM NADPH, DL-glyceraldehyde (5–25 mM), 5 μL of AR at a concentration of 100 μg/mL, and 100 μL of leaf extract (25, 50, and 75 μg/mL in DMSO less than 1%) in a total volume of 1.0 mL of 100 mM sodium phosphate buffer at a pH of 6.2. Within a separate set of tubes, the leaf extract was substituted with 100 μL of DMSO, which functions as the control. The absorbance change was quantified at a wavelength of 340 nm and then transformed into reaction velocities. A Lineweaver-Burk plot was generated to ascertain the mechanism of inhibition.

### Assessment of SD inhibition

The determination of SD activity was conducted following the methodology outlined in previous studies (Kazeem et al., 2020; Kobayashi et al., 2002). The assay combination consisted of a 100 mM Tris-HCl buffer with a pH of 9.0, 0.5 mM NAD+, 50 μL of SD at a concentration of 100 μg/mL, 100 mM sorbitol as the substrate, and a methanolic extract ranging from 12.5–100 μg/mL (in DMSO less than 1%). The reaction commenced with the introduction of NAD+. The absorbance transition rate of the combination was quantified at 340 nm using a BioSpectrometer (Eppendorf, United States). Quercetin was in addition employed as a positive control. The percentage of inhibition of sorbitol dehydrogenase was calculated using the following equation:

$$\%\text{ Inhibition}=\left[\left(\Delta \text{Abscontrol}-\Delta \text{Absextract}\right)÷\;\Delta \text{Abscontrol}\right]\times 100$$

where ΔAbs_control_ and ΔAbs_extract_ represent change in the absorbance of control (DMSO only) and leaf extract respectively.

### Analysis of the SD kinetics

The inhibition mechanism of SD by the leaf extract was carried out following the previously published studies with slight adjustments (Kazeem et al., 2020; Lindstad et al., 2013). The reaction mixture in one set of tubes consisted of a 100 mM tris-HCl buffer with a pH of 9.0, 0.5 mM NAD+, 50 μL of SD at a concentration of 100 μg/mL, sorbitol ranging from 50 to 250 mM, and methanolic extract solutions at concentrations of 25, 50, and 75 μg/mL (in DMSO less than 1%). In a separate pair of tubes, the leaf extract was substituted with DMSO alone, serving as the control. The absorbance change was quantified at a wavelength of 340 nm and then transformed into reaction velocities. Lineweaver-Burk plot was generated to ascertain the inhibitory mechanism.

### Determination of antiglycation activity

The determination of antiglycation activity was conducted using a method previously published by Hwang et al. (2018). Bovine serum albumin (BSA) at a concentration of 50 mg/mL was mixed with methylglyoxal (100 mM) in a sodium phosphate buffer (0.1 M, pH 7.4) while different amounts of the methanolic leaf extract were added. The mixture was then incubated at 37°C for 24 h. Next, the fluorescence intensity was quantified using a Cary Eclipse Fluorescence Spectrophotometer from Agilent Technologies. The excitation wavelength was set at 355 nm and the emission wavelength at 460 nm.

### *In vitro* quantification of sorbitol in red blood cells (RBCs)

The isolation and incubation of RBCs were carried out according to the method described by Malone et al. (1984). Blood samples of 5 mL were obtained from healthy male volunteers following an overnight fasting period and placed into heparinised tubes. RBCs were separated by centrifugation and then rinsed with isotonic saline at 4°C. RBCs were reconstituted in Kreb’s-ringer bicarbonate buffer at pH 7.4, which had been pre-equilibrated with 5% carbon dioxide. The duplicate samples were subjected to incubation at 37°C for 3 h in the presence of 5% CO_2_, under both normal (5.5 mM) and high glucose (55 mM) conditions. The accumulation of sorbitol was assessed by exposing the RBCs to different amounts of leaf extract.

Upon completion of the incubation period, RBCs were pulverised in nine volumes of 0.8 M perchloric acid. The homogenate was subjected to centrifugation at 5,000 g at 4°C for 10 min. The pH of the supernatant was then modified to 3.5 by adding a 0.5 M potassium carbonate solution. The sorbitol concentration in the supernatant was quantified using a fluorometric technique as described in other studies (Malone et al., 1980; Nagasaka et al., 1988).

### Statistical analysis

Except as otherwise specified, all analyses were conducted in triplicate. Data were reported as the mean ± standard error of the mean (SEM) and statistical significance was defined as a *p*-value less than 0.05. The statistical analysis was conducted using one-way ANOVA.

## Results

### Inhibition of polyol enzymes by *Ducrosia anethifolia* methanolic leaf extract

The methanolic extract of *Ducrosia anethifolia* exhibited a dose-dependent inhibitory effect on AR, with an IC50 value of 69.41 ± 3.59 μg/mL. Furthermore, quercetin, employed as a positive control, exhibited an IC50 value of 12.21 ± 4.13 μg/mL against AR. Table 1 presents the inhibitory activity of the methanolic extract against AR. Likewise, the methanolic extract was found to inhibit SD activity in a dose-dependent manner, with an IC50 of 31.11 ± 5.58 μg/mL. Quercetin, which served as the positive control, had an IC50 of 8.14 ± 2.27 μg/mL. The inhibitory property of methanolic extract against SD is presented in Table 2.

**TABLE 1:** AR inhibitory activity of methanolic extract of *Ducrosia anethifolia*.

	Concentration (µg/mL)		Inhibition (%)	IC_50_ (µg/mL)
AR activity	*D. anethifolia* extract (Methanolic)	200	92.78 ± 3.95^c^	69.41 ± 3.59
		100	64.67 ± 2.99^c^	
		50	40.09 ± 4.00^b^	
		25	25.22 ± 3.99^a^	
		12.5	14.36 ± 1.96^a^	
	Concentration (µg/mL)		Inhibition (%)	IC_50_ (µg/mL)
	Quercetin	40	82.49 ± 3.59^c^	12.21 ± 4.13
		20	59.28 ± 2.71^c^	
		10	39.06 ± 3.71^b^	
		5	22.61 ± 4.36^a^	
		2.5	9.73 ± 2.51^a^	

Where, a < 0.05, b < 0.01 and c < 0.001 (the significant difference as compared to control group).

**TABLE 2:** SD inhibitory activity of methanolic extract of *Ducrosia anethifolia*.

	Concentration (µg/mL)		Inhibition (%)	IC_50_ (µg/mL)
SD activity	*Ducrosia anethifolia* extract (Methanolic)	100	78.56 ± 4.51^c^	31.11 ± 5.58
		50	58.07 ± 3.66^c^	
		25	36.22 ± 4.54^b^	
		12.5	20.71 ± 3.11^a^	
		6.25	10.14 ± 2.08^a^	
	Concentration (µg/mL)		Inhibition (%)	IC_50_ (µg/mL)
	Quercetin	40	85.72 ± 2.37^c^	8.14 ± 2.27
		20	74.42 ± 3.20^c^	
		10	59.42 ± 3.11^c^	
		5	35.46 ± 3.21^b^	
		2.5	15.86 ± 2.32^a^	

Where, a < 0.05, b < 0.01 and c < 0.001 (the significant difference as compared to control group).

### Mixed-type inhibition of AR and SD enzymes by *Ducrosia anethifolia* methanolic leaf extract

**FIGURE 1:**
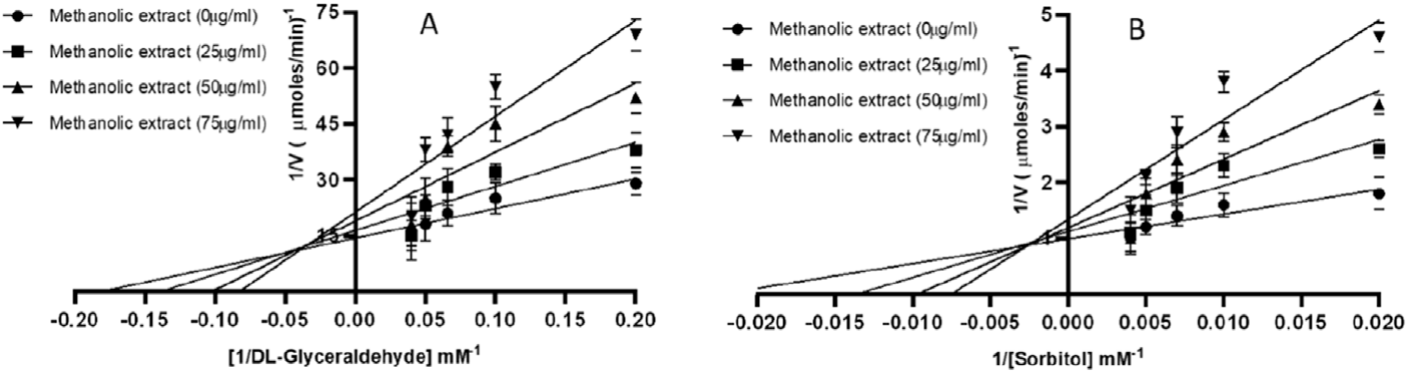
Mixed inhibition of **(A)** AR **(B)** SD by *Ducrosia anethifolia* methanolic extract.

The Lineweaver-Burk plots demonstrate that the methanolic extract exerts mixed inhibition on both AR and SD enzymes (Table 3; Figure 1). Table 3 demonstrates that the maximum velocities (Vmax) and Michaelis constant (Km) of both AR and SD enzymes vary as the extract concentration increases from 25 to 75 μg/mL, suggesting a mixed inhibitory relationship.

**TABLE 3:** Enzyme kinetics for the inhibition of AR and SD by methanolic extract of *Ducrosia anethifolia*.

Enzymes	Concentration (µg/mL)	K_m_ (mM)	V_max_ (µM/min)
AR	0	5.51	0.069
	25	7.20	0.061
	50	9.77	0.053
	75	12.08	0.047
SD	0	44.72	1.01
	25	73.21	0.89
	50	103.15	0.84
	75	132.89	0.75

### Inhibition of AGEs formation by *Ducrosia anethifolia* methanolic leaf extract

The methanolic extract of *Ducrosia anethifolia* was further employed to assess its impact on the production of AGEs. Furthermore, the results indicated a concentration-dependent suppression of methylglyoxal-BSA glycation in an *in vitro* condition, with an IC50 value of 310.00 ± 4.65 μg/mL (Table 4). Furthermore, aminoguanidine, employed as a positive control, exhibited an IC50 value of 193.9 ± 5.72 μg/mL.

**TABLE 4:** Antiglycation activity of methanolic extract of *Ducrosia anethifolia*.

	Concentration (µg/mL)		Inhibition (%) of AGEs formation	IC_50_ (µg/mL)
Antiglycation Activity	*Ducrosia anethifolia* extract (Methanolic)	1,000	86.43 ± 2.60^c^	310.00 ± 4.65
		500	64.12 ± 4.77^c^	
		250	42.95 ± 3.82^b^	
		125	25.22 ± 3.70^a^	
		62.5	14.49 ± 1.98^a^	
	Concentration (µg/mL)		Inhibition (%) of AGEs formation	IC_50_ (µg/mL)
	Aminoguanidine	500	91.64 ± 5.99^c^	193.9 ± 5.72
		250	55.97 ± 3.61^b^	
		125	38.93 ± 3.14^b^	
		62.5	29.26 ± 3.75^a^	
		31.25	16.09 ± 3.42^a^	

Where, a < 0.05, b < 0.01 and c < 0.001 (the significant difference as compared to control group).

### Reduction in the sorbitol accumulation by *Ducrosia anethifolia* methanolic leaf extract

The incubation of RBCs in a high glucose environment resulted in a more than three-fold rise in sorbitol concentration compared to the normal control. Conversely, the introduction of methanolic extract under this condition caused a concentration-dependent decrease in intracellular sorbitol (Table 5). This *in vitro* investigation confirmed the suppression of AR by the leaf extract.

**TABLE 5:** Effect of methanolic extract of *Ducrosia anethifolia* on sorbitol accumulation in RBCs.

Experimental groups	Sorbitol concentration (nmol/mL)
Normal glucose control	12.45 ± 4.21
High glucose control	42.61 ± 3.54^a^
High glucose + 50 μg/mL (methanolic extract)	35.93 ± 4.73^b^
High glucose + 100 μg/mL (methanolic extract)	26.24 ± 3.39^c^
High glucose + 200 μg/mL (methanolic extract)	13.94 ± 2.75^d^ ^,^ ^e^

^a^ <0.001; the significant difference between normal glucose control and high glucose control group.

^b^ <0.05; the significant difference between high glucose control and high glucose control + 50 μg/mL group.

^c^ <0.01; the significant difference between high glucose control and high glucose control + 100 μg/mL group.

^d^ <0.001; the significant difference between high glucose control and high glucose control + 200 μg/mL group.

^e^ >0.05; the significant difference between normal glucose control and high glucose control + 200 μg/mL group.

Generally, the findings of this investigation indicate that the methanolic leaf extract of *Ducrosia anethifolia* can be recommended for further progress in later phases of drug development due to its ability to inhibit AR and SD, as well as protein glycation, *in vitro*. Furthermore, the reduction in sorbitol accumulation in a high glucose environment further indicates the possible application of phytoconstituents of *Ducrosia anethifolia* in the treatment of diabetic retinopathy and other vascular problems. Hence, this work has the potential to make a substantial contribution in the area by providing powerful natural alternative treatment choice in the form of *Ducrosia anethifolia* phytoconstituents for the management of diabetes mellitus, particularly considering the negative consequences associated with synthetic inhibitors.

## Discussion

Diabetes mellitus is a chronic condition characterised by vascular disabilities, and many theories have been suggested to explain its development. Among these include the production of AGEs, accelerated rate of the hexosamine pathway, stimulation of protein kinase C, and enhanced flow of the polyol pathway (Dănilă et al., 2024). Numerous diabetes problems, such as cataract and kidney disease, have been linked to the aberrant polyol pathway (Simó et al., 2022; Wang and Lo, 2018). Previous studies have indicated that the suppression of AR and SD is a successful approach for decreasing the flow of the polyol pathway (Jannapureddy et al., 2021).

The present study was aimed to assess the inhibitory effect of methanolic leaf extract of *Ducrosia anethifolia* on the activities of AR and SD. While the IC50 of the methanolic extract was higher than that of quercetin; however, the lower IC50 value of quercetin can be ascribed to its purity. AR is the most crucial enzyme among the two because it catalyzes the rate-limiting step in the process (Chang et al., 2019). A low IC50 value was also observed for the inhibitory effect of the extract on SD. The results indicate that the extract from *Ducrosia anethifolia* has the potential to inhibit the accumulation of both sorbitol and fructose, hence inactivating the polyol pathway.

The rationale for choosing quercetin as the standard is based on its well-established role as a potent natural, plant derived antioxidant and its documented inhibitory effects on key pathways involved in diabetic retinopathy, such as the polyol pathway (Varma et al., 1975; Chaudhry et al., 1983). Quercetin has been widely used in the literature as a positive control due to its recognized therapeutic potential in mitigating oxidative stress, inflammation, and other complications related to diabetes (Yasir et al., 2024; Goodarzi et al., 2006). It serves as an appropriate benchmark for comparing the efficacy of *Ducrosia anethifolia* extract in inhibiting the enzymes AR and SD, which are critical in the development of diabetic retinopathy. Furthermore, quercetin is a known flavonoid with various beneficial effects in the context of diabetic complications, and its use allows for comparison with the extract in terms of both potency and mechanisms of action.

Given its potent suppression of AR and SD, we performed the kinetics of enzyme inhibition by the methanolic leaf extract. The double reciprocal plot demonstrated that the extract could inhibit both AR and SD enzymes via mixed-inhibition. This observation implies that active component(s) of the leaf extract may possess functional group(s) that exhibit significant affinity for both the active sites and other binding sites of the enzymes. Notably, a recent study has demonstrated that mixed inhibitors specifically attach to the active region of enzymes (Pesaresi, 2023). While the exact active compounds in *Ducrosia anethifolia* extract have not been isolated in this study, bioactive compounds such as coumarins, terpenoids and flavonoids, are known to possess functional groups that may interact with key enzymes, such as AR and SD, involved in the polyol pathway. However, to confirm binding interactions and identify specific binding sites, further studies, including molecular docking or enzyme inhibition assays, would be necessary. Although we understand that further in-depth *in vitro* and *in vivo* study is required to establish this fact, our preliminary results at least set a ground for further exploration of *Ducrosia anethifolia* extract for the identification of individual phytochemicals responsible for the strong inhibition of polyol enzymes.

Conventionally, the process of converting glucose to sorbitol leads to a reduction of NADPH, which is essential for the restoration of reduced glutathione, thereby inducing oxidative stress (Hwang et al., 2018). Furthermore, NAD + serves as the cofactor for SD in the synthesis of fructose, therefore causing a redox imbalance. Cellular osmotic stress and the production of AGEs are induced by the buildup of sorbitol and fructose, respectively (Rungratanawanich et al., 2021). The methanolic extract can enhance the mitigation of oxidative stress, osmotic stress, and glycation associated with this process by inhibiting both AR and SD activities simultaneously.

Prior investigations have demonstrated that the activation of AR in RBCs results in an elevated buildup of sorbitol (Srivastava et al., 2011; Kheirollah et al., 2015). An analogous investigation revealed a clear and direct relationship between the levels of erythrocyte AR and sorbitol (Akileshwari et al., 2012). The findings of our study indicate a notable decrease in the deposition of sorbitol in RBCs when the dosage of *Ducrosia anethifolia* leaf extract is increased. Here, we want to acknowledge that 55 mM glucose concentration is indeed much higher than typical hyperglycemic levels (e.g., 10–25 mM glucose) observed in physiological conditions. The choice of 55 mM glucose was based on its widespread use in *in vitro* models as an extreme hyperglycemic condition to induce robust and measurable changes in biochemical pathways, such as the polyol pathway, oxidative stress, and protein glycation. This approach is commonly employed to simulate diabetic complications like diabetic retinopathy and cataract formation, and observe the therapeutic effects of interventions under exaggerated stress conditions (Katta and Murugan, 2022; Aware et al., 2021; Rajagopala et al., 2020; Ganeshpurkar et al., 2011). Moreover, Nagasaka et al. (1988) have measured the sorbitol accumulation in RBCs by incubating them with a range of 5–50 mM glucose concentration (Nagasaka et al., 1988). Although, 500 mg/dL glucose equivalent to 27.8 mM have been commonly used for studying sorbitol accumulation in RBCs (Malone et al., 1984; Malone et al., 1980). We recognize that such concentrations may not fully reflect *in vivo* scenarios, but to mimic extreme hyperglycemic conditions that are often present in diabetic patients, particularly in the context of uncontrolled or poorly managed diabetes (and diabetic retinopathy and cataract formation like situation), we utilized this much higher concentration of glucose to study sorbitol accumulation and its mitigation by *Ducrosia anethifolia* extract. Furthermore, the leaf extract of *Ducrosia anethifolia* shown a dose-dependent suppression of AGEs production, indicating its potential role in antiglycation and ultimately, minimizing the menace of debilitating AGEs formation in diabetic retinopathy.

Shalaby et al. (2014) have documented *Ducrosia anethifolia* as a substantial reservoir of coumarins, including psoralen, 5-methoxypsoralen, 8-methoxypsoralen, imperatorin, isooxypeucedanin, pabulenol, oxypeucedanin methanolate, and oxypeucedanin hydrate. Stavri et al. (2003) identified a furanocoumarin pangelin and a monoterpene glucoside 8-debenzoylpaeoniflorin from the aerial part of *Ducrosia anethifolia*. The presence of these secondary metabolites may be ascribed to their antioxidant, anticancer, anti-inflammatory, and antidiabetic effects (Shahabipour et al., 2013). There are a limited number of studies that have shown the antidiabetic and hypoglycemic effects of *Ducrosia anethifolia* (Shalaby et al., 2014; Syed et al., 2022; Jahromi et al., 2024). Thus, the findings of this study have the potential to establish a novel foundation for the management of diabetic retinopathy and other vascular complications.

Despite the above-described significant findings, the current study is limited by only emphasizing the potential of the extract, however, identifying compounds is an important step in any drug development process, therefore, future research could be integrated with various chromatographic analysis technique to characterize the methanolic extract of *Ducrosia anethifolia*. This will allow to identify the major bioactive compounds present in the extract. Moreover, following the identification of key phytoconstituents, molecular docking studies can be performed to predict the interactions of these compounds with key enzymes in the polyol pathway along with *in vitro* inhibition assay of these enzymes to validate the molecular docking results. Moreover, potency of the extract can be improved by isolating and concentrating the most active phytoconstituents, optimizing extraction methods, or synthesizing derivatives of the active compounds with enhanced bioactivity. While logistical constraints currently limit our ability to perform further *in vitro* and *in vivo* experiments, we acknowledge their importance which could provide insights into the extract’s efficacy and safety profile in a physiological context.

## Conclusion

It can be concluded that *Ducrosia anethifolia* methanolic extract displayed potent inhibition of the activities of polyol pathway enzymes. A mixed-inhibition mechanism was seen in the activity of polyol pathway enzymes by the methanolic extract of *Ducrosia anethifolia*. Furthermore, the production of AGEs and the accumulation of sorbitol in RBCs were also significantly reduced. Thus, our experimental findings indicate that the methanolic extract of *Ducrosia anethifolia* has the ability to suppress enzymes involved in the polyol pathway, which may contribute to ameliorate diabetic retinopathy and hence the disability associated with it, including vision disability. The observed phenomena may be linked to the existence of many phytochemicals in *Ducrosia anethifolia* extract. Nevertheless, additional investigation is necessary to identify the specific chemicals that are accountable for the observed activity.

## Data Availability

The original contributions presented in the study are included in the article/supplementary material, further inquiries can be directed to the corresponding author.
